# Supplementary material for: The causal relationship between the human gut microbiota and pyogenic arthritis: a Mendelian randomization study
Source: Front Cell Infect Microbiol. 2024 Nov 26;14:1452480. doi: 10.3389/fcimb.2024.1452480 (PMC11629706; doi:10.3389/fcimb.2024.1452480)
Supplement: Supplementary file 1 [file DataSheet1.zip › mendelian test/ebi-a-GCST90027594.csv_forest.pdf]

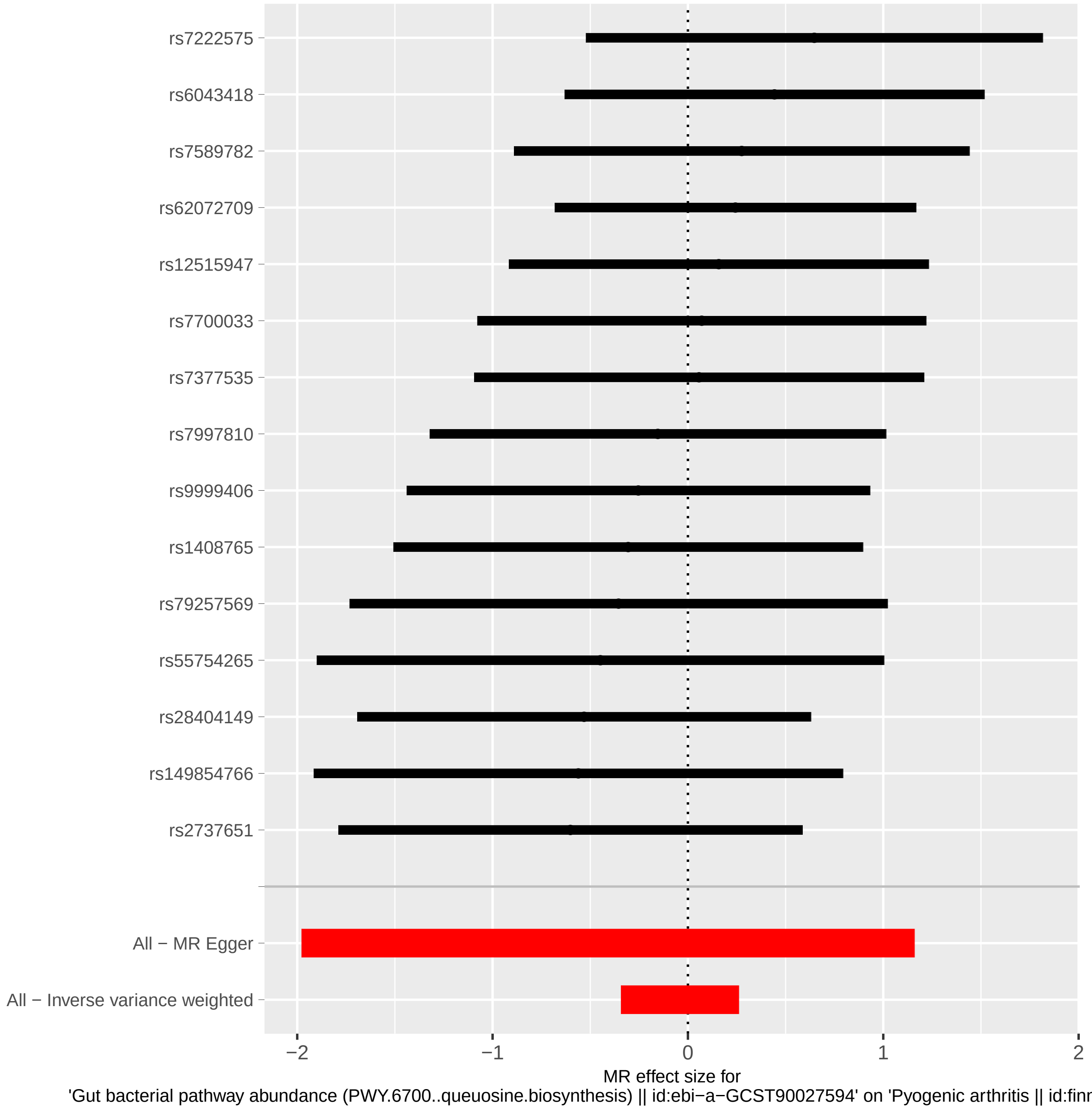
rs7222575
rs6043418
rs7589782
rs62072709
rs12515947
rs7700033
rs7377535
rs7997810
rs9999406
rs1408765
rs79257569
rs55754265
rs28404149
rs149854766
rs2737651
All − MR Egger
All − Inverse variance weighted
−2
−1
0
1
2
MR effect size for
'Gut bacterial pathway abundance (PWY.6700..queuosine.biosynthesis) || id:ebi−a−GCST90027594' on 'Pyogenic arthritis || id:finn
